# Acute sub-diaphragmatic anterior vagus nerve stimulation increases peripheral glucose uptake in anaesthetized rats

**DOI:** 10.1016/j.ibneur.2023.06.005

**Published:** 2023-06-17

**Authors:** C.W. Hoornenborg, T.H. van Dijk, J.E. Bruggink, A.P. van Beek, G. van Dijk

**Affiliations:** a Groningen Institute for Evolutionary Life Sciences (GELIFES), Department of Behavioral Neuroscience, University of Groningen, Groningen, the Netherlands; b Department of Endocrinology, University of Groningen, University Medical Center Groningen, Groningen, the Netherlands; c Department of Laboratory Medicine, University of Groningen, University Medical Center Groningen, Groningen, the Netherlands

**Keywords:** Glucose fluxes, Metabolic diseases, Vagus nerve stimulation, Norepinephrine

## Abstract

The sub-diaphragmatic vagus innervates various organs involved in the control of glucose homeostasis including the liver, pancreas and the intestines. In the current study, we investigated the effect of acute electrical stimulation of the anterior trunk of the sub-diaphragmatic vagus on glucose fluxes in anaesthetized adult male rats.

After overnight fast, rats underwent either vagus nerve stimulation (VNS+, n = 11; rectangular pulses at 5 Hz, 1.5 mA, 1 msec pulse width) or sham stimulation (VNS-; n = 11) for 120 min under isoflurane anesthesia. Before stimulation, the rats received an i.v. bolus of 1 mL/kg of a sterilized aqueous solution containing 125 mg/mL of D-[6,6-^2^H_2_] glucose. Endogenous glucose production (EGP) and glucose clearance rate (GCR) were calculated by kinetic analysis from the wash-out of injected D-[6,6–^2^H_2_]glucose from the circulation.

VNS+ resulted in lower glucose levels compared to the VNS- group (p < 0.05), with similar insulin levels. EGP was similar in both groups, but the GCR was higher in the VNS+ group compared to the VNS- group (p < 0.001). Circulating levels of the sympathetic transmitter norepinephrine were reduced by VNS+ relative to VNS- treatment (p < 0.01).

It is concluded that acute anterior sub-diaphragmatic VNS causes stimulation of peripheral glucose uptake, while plasma insulin levels remained similar, and this is associated with lower activity of the sympathetic nervous system.

## Highlights

• The sub-diaphragmatic vagus innervates various organs involved in glucose homeostasis.
• Vagal nerve stimulation is an effective treatment for several clinical conditions.
• Anterior sub-diaphragmatic vagus nerve stimulation increased peripheral glucose uptake without affecting insulin levels.
• Sub-diaphragmatic vagus nerve stimulation may be of potential in treatment of diseases associated with sympathetic overload.

## 1 Introduction

Vagus nerve stimulation (VNS) has recently been approved by the Food and Drug Administration (FDA) as treatment option for epilepsy (1997), depression (2005) and obesity (2015), and is currently getting more attention as potential new treatment against type 2 diabetes (T2DM) (Clancy et al., 2013; Malbert, 2018). Abdominal organs involved in food intake and glucose homeostasis, including liver, pancreas and the intestines are innervated by efferent parasympathetic fibers within the three abdominal (hepatic, gastric and celiac) branches of the vagus nerve (Berthoud and Neuhuber, 2000a, Berthoud and Neuhuber, 2000b). In addition, branches of the vagus nerve contain afferent fibers from the abdominal organs to the brain that signal various aspects of nutritional and gastrointestinal processes (Berthoud, 2008). After a meal, elevated glucose entry from the intestines could be detected by hepatic portal glucose sensors, which initiates a signal to the brain via afferent nerves promoting satiety (Mithieux, 2014), as well as a reflex mechanism that might control hepatic glucose fluxes accordingly (Niijima, 1983). Efferent parasympathetic activity may play a role in the latter process, as cholinergic blockade by atropine reduces hepatic glucose uptake after an oral glucose load in dogs (Chap et al., 1985). Thus, the vagus nerve is an integral part of feeding behaviour and glucose homeostasis (Payne et al., 2018). The balance of parasympathetic activity versus sympathetic activity is often shifted towards the latter in conjunction to various cardiometabolic derangements including chronic inflammatory disorder, hypertension, glucose intolerance and insulin resistance (Bonaz et al., 2017, Hurr et al., 2019). Electrical stimulation of specific vagal trunks in cardiometabolic diseases could potentially shift back the autonomic disbalance. Payne et al. showed that sub-diaphragmatic vagus nerve stimulations with different electrical stimulation strategies modulates glycemia by affecting glucagon and insulin secretion (Payne et al., 2020). In a study by Malbert et al., it was shown that sub-diaphragmatic bilateral VNS in minipigs induces an increase in whole-body glucose uptake and improved insulin sensitivity (Malbert et al., 2017). Yin et al. showed that electrical stimulation of the posterior sub-diaphragmatic vagus trunk improved glucose homeostasis in T2DM rats presumably depending on incretin actions involving GLP-1 (Yin et al., 2019). However, contrasting effects on circulating glucose and insulin levels are also seen in different studies after vagus nerve stimulation (Dai et al., 2020, Meyers et al., 2016, Stauss et al., 2018), which could be attributed to stimulation at different locations along the vagus nerve. For example, Meyers et al. found that cervical VNS caused severe and sustained hyperglycemia without stimulating insulin secretion (Meyers et al., 2016). Effects of VNS selectively on the anterior sub-diaphragmatic vagus trunk on glucose homeostasis is currently lacking. For that purpose we analyzed the effects of anterior sub-diaphragmatic VNS on glucose homeostasis by using a labelled glucose technique, allowing kinetic assessment of glucose fluxes in the body and tissue-specific glucose dynamics, and we assessed plasma levels of insulin and catecholamines.

## 2 Material and methods

### 2.1 Animals

The animal experiment and procedures complied with the principles of laboratory animal care following the EU-directive for the protection of animals used for scientific purposes. All animal procedures were approved by the Dutch Central Committee on Animal Experimentation (license number AVD1050020186284) and were performed in accordance with the NIH Guide for the Care and Use of laboratory Animals.

Male Wistar rats (566.3 ± 9.0 g, n = 12) aged 8–12 months were group housed in cages filled with wood chip bedding and a gnawing stick with ad libitum rat chow and water. Climate was controlled at 20 ± 2 °C, humidity at 60 ± 5% and the light-dark cycle was 12:12 h (lights ON at 09:00 AM).

### 2.2 Instrumentation

**Fig. 1:**
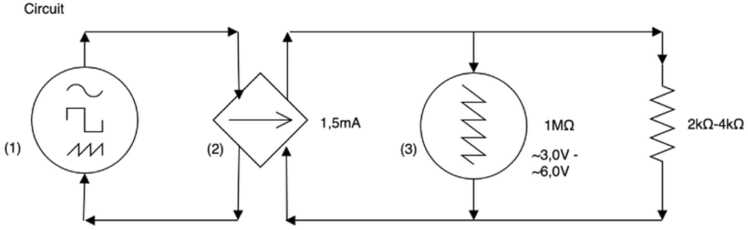
Schematic overview of the electrical circuit. The pulse generator (1) created a monophasic pulse of 5 Hz and a width of 1 ms. The constant current unit (2) ensures a constant current of 1,5 mA. The oscilloscope (3) was used to monitor the pulse.

An electrical stimulator (Grass Medical Instrument SD9) was used to produce a voltage with a set pulse width and pulse frequency. To ensure a constant current of 1.5 mA, a constant current unit was used (CCU1A). To validate and control if the pulse was transmitted properly, a Grass Tektronix oscilloscope 2225 was used (Fig. 1). Based on Ohm’s law: V=I×R, where current was 1.5 mA and resistance (between 2 kΩ and 4 kΩ (Groves and Brown, 2005)), the electrical pulse should generate a voltage between 3 and 6 V (read-out of the oscilloscope). The stimulation electrodes were constructed in an adapted form from the protocol of Childs et al. In short, two stainless steel wires (Ø 0.2 mm) were fed through polyethylene tubes and placed and secured inside a silicone tube (cuff; Ø 3 mm outside, Ø 1 mm inside) ± 3.5 mm in length (Childs et al., 2015).

### 2.3 Experimental protocol

Before the experimental day, food was removed 12 h before the start of the experiment (fasting period 22:00–10:00). After fasting, body weight of rats to be subjected to VNS (VNS+; 547.7 g±22.2) was similar compared to the control group (VNS-; 548.5 g±10.6).

All surgical procedures were performed under isoflurane anesthesia (5% induction, 2% maintenance) while the rats were kept warm on a heating pad. First, a silicon catheter was inserted in the jugular vein, allowing remote sampling from the right atrium of the heart (Strubbe, 1977).

**Fig. 2:**
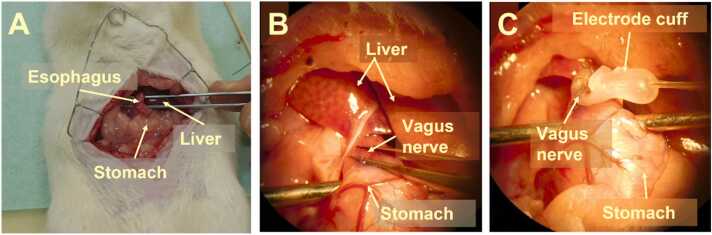
Surgical procedure for the attachment of the electrodes to the vagus nerve. A) An open abdominal view, with the esophagus, liver and stomach exposed. The anterior vagus nerve can be located in the connective tissue surrounding the esophagus. B) Depicts the exposed vagus nerve identified by adding 1% toluidine blue and detached from the surrounding tissue. C) Depicts the exposed vagus nerve in the electrode cuff.

After the jugular vein cannulation, the abdomen was shaved and disinfected with surgical scrub, followed by midline laparotomy. The sub-diaphragmatic anterior vagus nerve (innervates both the liver and the pancreas (Berthoud and Neuhuber, 2000a, Berthoud and Neuhuber, 2000b)) was identified by gently adding a small amount of 1% toluidine blue solution to the sub-diaphragmatic esophagus (Arthur and Shelly, 1959). Then, the anterial vagus nerve trunk was gently detached from the esophagus after which the cuff was placed around it, and secured into place using a suture. The electrode leads were attached to the stimulator (VNS+; n = 11) or left unattached (VNS-; n = 11). Electrical stimulation (rectangular pulses at 5 Hz, 1.5 mA, 1 msec pulse width was initiated and maintained for 120 min in the VNS+ group (Meyers et al., 2016, Pelot and Grill, 2018, Stauss, 2017), and the VNS- group did not receive any electrical stimulation. For an overview of the surgical procedure see Fig. 2.

### 2.4 Blood sampling and tracer injection

Rats received an i.v. bolus of 1 mL/kg of a sterilized aqueous solution containing 125 mg/mL of D-[6,6-^2^H_2_]glucose (Sigma-Aldrich) just before the start of vagus nerve stimulation or sham stimulation. Blood samples (500 µL) were taken via the jugular catheter at time-points − 15, 0, 30, 60, 90 and 120 after the start of vagus nerve or sham stimulation for analysis of blood glucose, plasma insulin, and catecholamines. Additionally, blood spots were taken on filter paper (Sartorius stedim TNF 180 g/m2, Nieuwegein, the Netherlands) at time-points − 15, 15, 30, 45, 60, 90 and 120 min after the start of vagus nerve or sham stimulation for GC-MS analysis. For this, the spots were air-dried and stored at room temperature until further analysis. Blood samples were immediately put on ice in vials containing 10 µL EDTA (0.09 g/mL). Whole blood samples of 50 µL were diluted in 450 µL 2% heparin solution and stored at − 20 °C until analysis of glucose concentrations by the ferry-cyanide method in a Technicon auto-analyzer (Evers et al., 2017). The remaining blood samples (450 µL) were centrifuged (15 min, 2500 rpm, 4 °C) and plasma was collected and stored at − 20 °C until insulin determination ( ± 100 µL). Plasma insulin levels were measured using a commercial radioimmunoassay kit (Rat Insulin, I-Insulin Cat# RI-13 K, Linco Reasearch, Nucli Lab, The Netherlands). Plasma for catecholamine analysis was collected (±200 µL) and stored at − 80 °C until further analysis.

### 2.5 Derivatization and GC-MS measurements of glucose

The fractional distribution of D-[6,6-^2^H_2_]glucose in the dried bloodspots were measured according to van Dijk et al. (van Dijk et al., 2013). In short, glucose was extracted from bloodspots by punching out a disk (6.5 mm in diameter), transferred to a reaction vial and wetted with 50 µL water for 10 min followed by the addition of 500 µL ethanol after which the mixture was incubated overnight at room temperature. Subsequently, the solution was centrifuged for five minutes after which 400 µL supernatant was removed and transferred to a Teflon-capped reaction vial and dried at 60 °C under a stream of nitrogen. Glucose was converted to its pentaacetate derivative by adding 300 mL pyridine/acetic anhydride (1:2) to the residue, incubating it overnight at room temperature. After drying at 60 °C under a stream of nitrogen the residue was dissolved in 200 µL ethyl acetate and transferred into injection vials for analysis. All samples were analyzed by gas chromatography mass spectrometry (Agilent 9575 C inert MSD; Agilent Technologies, Amstelveen, the Netherlands). Mass spectrometric analyses were performed by positive chemical ionization with ammonia. Ions monitored, *m/z* 408–414 (*m*_*0*_ - *m*_*6*_), were corrected for the fractional distribution due to natural abundance of ^13^C by multiple linear regression as described by Lee et al. (Lee et al., 1991) to obtain the excess fractional distribution of mass isotopologues (*M*_*0*_ – *M*_*6*_ with $\sum_{i=0}^{i=6}\;M_{i}=1$ ) due to the dilution of administered D-[6,6-^2^H_2_]glucose, i.e*.* M_2_ represents the fractional contribution of D-[6,6-^2^H_2_]glucose in blood glucose and was used in the calculations of blood glucose kinetics (van Dijk et al., 2013).

### 2.6 Calculation of blood glucose kinetics

We used a single-pool first-order kinetic model for basal glucose kinetics as described before (Ader et al., 1997, Dube et al., 2015, van Dijk et al., 2013). Essentially, calculations were performed as presented by van Dijk et al. However, for this specific experiment, i.e*.*, two different experimental periods with in one tracer elimination curve, the equations had to be adjusted somewhat as shown in supplementary table 1. First the glucose clearance rate (GCR) was calculated from the wash-out of injected D-[6,6-^2^H_2_]glucose from the circulation and the administered bolus. Next, the apparent volume of distribution (V) was calculated from the administered bolus and the estimated initial tracer concentration. This in combination with the blood glucose concentration resulted in the pool size of glucose (A). Finally, the endogenous glucose production (Ra) was calculated the product of pool size and fractional elimination rate (k). To estimate the kinetic parameters, a single-compartment model was implemented in SAAM II (SAAM II v2.3, The Epsilon Group, Charlottesville, VA, USA).

### 2.7 HPLC quantifications of catecholamine concentrations

Determination of plasma catecholamine concentrations was performed by high-pressure liquid chromatography (HPLC) in combination with electrochemical detection as described previously (Smedes et al., 1982), with some small modifications. The high-pressure liquid chromatography-electrochemical detection system (detector: Antec Decade, 2, VT-03 electrochemical flow cell) included a Shimadzu LC-10AD 2150 pump, and a Phenomenex Gemini 5 µm C_18_ 110 A, column (150 ×4.6 mm). The eluent contained 10.5 g/l citric acid monohydrate, 8.0 g/l Na_2_PO_4_ * 2 H_2_0, 0.3 g/l EDTA, 1 mL/l Methanol, 0.50 g/l Octane Sulfonic Acid and 999 mL Ultrapure H_2_O (pH 3.6). The flow was 1.5 mL/min and the temperature was held constant at 30 °C.

### 2.8 Statistical analyses

Within this experiment, two main effects on blood glucose kinetics were studied, i.e*.*, the intervention (VNS+; n = 9 and VNS-; n = 11) and the period of time (metabolic inactive period of VNS; between timepoints 15–45 min and metabolic active period of VNS between timepoints 60–120 min). For this, a two-way ANOVA was used (SPSS). All data are expressed as mean ± standard error of the mean (SEM).

## 3 Results

### 3.1 Glucose and insulin response

**Fig. 3:**
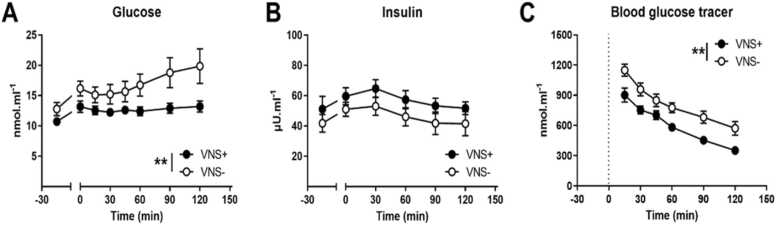
(A) Blood glucose, (B) plasma insulin and (C) blood glucose tracer during vagus nerve stimulation (VNS+, closed circles) or sham stimulation (VNS-; open circles. Data are shown as mean ± SEM. ** between the groups p < 0.01.

Average fasting glucose and insulin concentrations before the start of vagus nerve stimulation was similar in the VNS+ and VNS- group. However, two-way repeated ANOVA revealed a significant interaction of glucose with time (F(8,144) = 3.441, p < 0.01), with circulating glucose levels in the VNS+ group being significantly lower compared to the VNS- group (Fig. 3 A). In contrast, there was no effect of vagus nerve stimulation on plasma insulin levels (Fig. 3B). Blood glucose tracer levels decreased over time due to a time effect (F(5, 90) = 116.2 p<0.001). Between subjects analysis showed that the wash-out period of D-[6,6-^2^H_2_]glucose from the circulation system was significantly faster in the VNS+ group compared to the VNS- group ((F1, 18) = 8,399; p < 0.01; Fig. 3 C).

### 3.2 Glucose kinetics

**Fig. 4:**
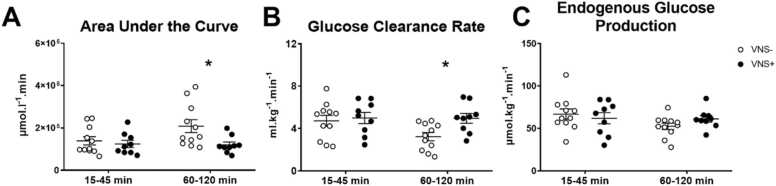
(A) The area under the curve of glucose, (B) the glucose clearance rate and (C) endogenous glucose production of the blood glucose tracer during the metabolic inactive (15–45 min) and metabolic active (60–120 min) phase of vagus nerve stimulation (VNS+; closed circles) or sham stimulation (VNS-; open circles). Data are shown as mean ± SEM. * shows an interaction effect of time (metabolic inactive versus metabolic active phase) and intervention (VNS+ versus VNS-) p < 0.05.

Post-hoc testing for sources of significance of blood glucose differences between groups showed that VNS+ caused lower blood glucose levels at time-points 60, 90 and 120 min, while levels until timepoint 45 min were not significantly different. Apparently a metabolic inactive (15–45 min) and metabolic active (60–120 min) period can be distinguished during VNS. There was a time effect (metabolic inactive versus metabolic active phase) on the area under the curve (AUC) of glucose tracer (F(1, 18) = 5.021; p < 0.05; Fig. 4 A). In addition, the AUC of glucose tracer showed a significant interaction effect with the intervention, where the VNS+ group was significantly lower compared to the VNS- group during the active period of VNS (F(1, 18) = 6.222 p < 0.05; Fig. 4 A). Glucose clearance rate (GCR) showed a significant time (F(1, 18) = 8.461; p < 0.01) and significant intervention×time effect. This resulted in a higher GCR during the metabolic active (but not during the metabolic inactive) phase in the VNS+ group compared to the VNS- group (F(1, 18) = 7.715, p < 0.05; Fig. 4B). The endogenous glucose production (EGP) was calculated as a product of the pool size and elimination rate, no time effect or differences between the VNS+ and VNS- group were seen regarding EGP (Fig. 4 C).

### 3.3 Autonomic Activity

**Fig. 5:**
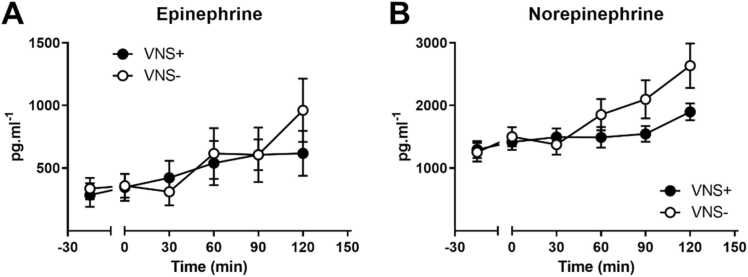
(A) Epinephrine, and (B) Norepinephrine during vagus nerve stimulation (VNS+ closed circles) or sham stimulation (VNS-; open circles). Data are shown as mean ± SEM.

Both circulating epinephrine (F(5, 90)= 0.8951+; p < 0.01) and norepinephrine (F(5, 90)= 2.303; p < 0.0001) levels increased over time during the experimental period, in both the VNS+ and VNS- group (Fig. 5). The latter (norepinephrine) showed a borderline significant interaction effect with the intervention (F(5, 90)= 2.303; p = 0.0511).

**Fig. 6:**
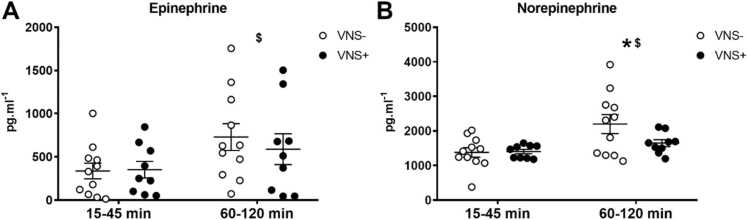
(A) Epinephrine, and (B) Norepinephrine during the metabolic inactive (15–45 min) and metabolic active (60–120 min) phase of vagus nerve stimulation (VNS+; closed circles) or sham stimulation (VNS-; open circles). Data are shown as mean ± SEM. $ p < 0.05 between time points; * p < 0.05 for an interaction between time (metabolic inactive versus metabolic active phase) and intervention (VNS+ versus VNS-).

When dividing the stimulation period in an inactive and active metabolic period, there was a time effect on epinephrine (F(1, 18)= 13.03; p < 0.01) and norepinephrine (F(1, 18)= 15.88; p < 0.001; Fig. 6). Moreover, the norepinephrine levels where significantly higher in the VNS- group during the metabolic period (60–120 min of stimulation) compared to the VNS+ group (F(1, 18)= 4.683; p < 0.05).

### 3.4 Relation of autonomic activity and glucose kinetics

**Fig. 7:**
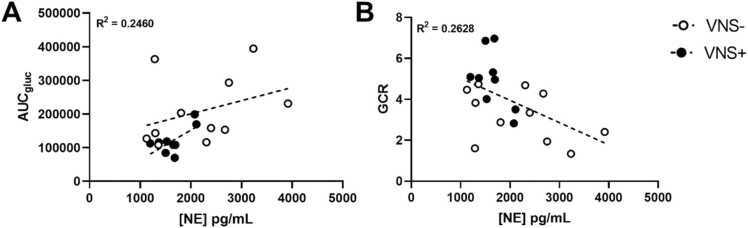
Correlation of (A) Area under the curve (AUC) of glucose, and (B) Glucose clearance rate (GCR) with norepinephrine (NE) during the metabolic active phase of vagus nerve stimulation (VNS+; closed circles) or sham stimulation (VNS-; open circles).

To investigate the effect of the autonomic activity during the metabolic active period (60–120 min) on the different glucose kinetic parameters, a linear regression analysis was performed. Norepinephrine was positively correlated with the AUC_gluc_ (B = 0.01674; r^2^ = 0.2460; p = 0.0261) irrespective of intervention (Fig. 7 A). When specifying for intervention, this positive correlation was seen in the VNS+ group (B = 0.01120; r^2^ = 0.4474; p = 0.0488) and not in the VNS- group (B= 0.02555; r^2^ = 0.1248; p = 0.2866). Furthermore, there was a negative correlation of norepinephrine with GCR (B = −0.00187; r^2^ = 0.2628 p = 0.0193, Fig. 7B) irrespective of intervention. Epinephrine levels did not show a correlation with any of the glucose kinetic parameters.

## 4 Discussion

The present study used stable isotopically-labelled glucose for analysis of glucose kinetics after stimulation of the anterior sub-diaphragmatic vagus nerve (VNS). We observed that the glucose-tracer level decreased faster in the VNS+ group, due to an increased glucose clearance rate (GCR), and without changes in endogenous glucose production (EGP). These effects are largely independent of insulin action as VNS did not show an effect on circulating insulin levels. Consequently, the GCR is probably dependent on an increased glucose uptake in peripheral tissue and not hepatic uptake, as the latter is insulin-dependent (Püschel, 2004). Important for consideration of these findings is that norepinephrine increased during the experiment (especially in the VNS- group), which could be effectively decreased in the VNS+ group. Reduced activity of the sympathetic branch of the autonomic nervous system directed towards the liver would be expected to result in reduced hepatic glucose production independent of insulin action (Ribeiro et al., 2015), which might explain our findings (although we cannot exclude other possibilities). Such a phenomenon could potentially be explained by reduction of norepinephrine release after activation of cholinergic receptors present on sympathetic nerve endings in the liver. This was already described in anesthetized dogs more than five decades ago (Levy and Blattberg, 1976, Lindmar et al., 1968), where VNS resulted in a 30% decrease of norepinephrine, which was prevented by the addition of atropine (Levy and Blattberg, 1976). Because acetylcholine is rapidly removed from the circulation (Levy and Blattberg, 1976), we can only speculate that VNS caused increased acetylcholine release from vagal nerve terminals acting on muscarinic receptors present on presynaptic sympathetic neurons, evoking this inhibitory effect on circulating norepinephrine levels (Trendelenburg et al., 2005). This effect could, for example, be the result of the M_3_-receptor, as M_3_-receptor deficient mice, with an increased sympathetic outflow, showed impaired glucose homeostasis and reduced insulin sensitivity (Gautam et al., 2008). However, the heterogeneity makes it difficult to pinpoint the specific (or combination of) muscarinic receptors responsible for the effects seen in this study and would it be of interest to study these in combination with vagus nerve stimulation.

Another or perhaps additional mechanism possibly responsible for the effect seen in this study could rely on electrical stimulation of vagal afferent branches giving feedback to the brain (Clancy et al., 2014, Fallen, 2005). In turn, the brain can influence glucose homeostasis by engaging insulin-independent mechanisms affecting both glucose disposal and production (Myers et al., 2021). The fact that we did not find an increase of plasma insulin levels in the VNS+ relative to the VNS- group may also be explained by findings in the study of Meyers et al., in which the stimulatory effect of efferent VNS on insulin release could be dissociated from the inhibitor effect of afferent VNS on insulin secretion (Meyers et al., 2016). Thus, projection of those findings to the outcomes of our study may potentially suggests that our VNS technique stimulated and inhibited insulin release through respectively activating efferent and afferent fibers, rendering the outcomes on glucose homeostasis in the present study insulin-independent.

A limitation in our study is the use of isoflurane as anesthetic agent. Although the isoflurane concentrations remained ≤ 2%, which should make the influence of the isoflurane anesthesia as small as possible (Constantinides et al., 2011; Desborough et al., 1993), there are studies that this could have influenced rates of glucose clearance, glucose turnover and tolerance (Sicardi et al., 2006). Even isoflurane in concentrations as low as 1% can impact on insulin secretion and sympathetic activity (Constantinides et al., 2011, Fitchett et al., 2021). Next to the use of isoflurane, the surgery-induced stress could also have had an effect on our results. Surgery-induced sympathetic activation of the liver can potently increase hepatic glucose production, especially in combination with the increased secretion of adrenaline and glucocorticoids from the adrenal glands (Myers et al., 2021). Despite these limitations, however, it is important to note that the VNS+ group as well as the VNS- group received exactly the same treatments (i.e., except of course for VNS itself), which yielded the clear outcome that VNS caused increased glucose disposal without affecting insulin levels. It does not seem very likely that these outcomes are solely dependent on the anesthetic condition, it may even be speculated that some of the effects of VNS were masked (e.g., because of behavioral components lacking in the anaesthetized condition). Our study should therefore be seen as a starting point for future studies on anterior sub-diaphragmatic VNS in unanesthetized animals under relevant conditions. These conditions could include the T2D rat model, like for instance has been employed in the study by Yin et al. (Yin et al., 2019), where posterior sub-diaphragmatic VNS was able to reduce glucose intolerance. In fact, our technique of anterior sub-diaphragmatic VNS resulting in increased glucose disposal combined with reduced sympathetic activity could complement the one employed by Yin et al. (Yin et al., 2019). Thus, for health complications in cardiometabolic diseases with shifted balance towards sympathetic overload (Bonaz et al., 2017, Carnethon et al., 2003, Hurr et al., 2019), sub-diaphragmatic VNS might offer therapeutic relief (Payne et al., 2020, Stakenborg et al., 2017).

## Funding

This research did not receive any specific grant from funding agencies in the public, commercial, or not-for-profit sector.

## CRediT authorship contribution statement

**C.W. Hoornenborg:** Conceptualization, Methodology, Software, Validation, Formal analysis, Investigation, Data curation, Writing – original draft preparation, Visualization, Project administration. **A.P. van Beek:** Conceptualization, Writing – review & editing, Supervision, Funding acquisition. **T.H. van Dijk:** Methodology, Software, Validation, Formal analysis, Investigation, Data curation, Writing – review & editing, Visualization. **J.E. Bruggink:** Methodology, Validation, Investigation, Writing – review & editing. **G. van Dijk:** Conceptualization, Resources, Writing – review & editing, Supervision, Funding acquisition.

## Data Availability

Data will be made available on request
