# Supplementary material for: Acute sub-diaphragmatic anterior vagus nerve stimulation increases peripheral glucose uptake in anaesthetized rats
Source: IBRO Neurosci Rep. 2023 Jun 17;15:50–6. doi: 10.1016/j.ibneur.2023.06.005 (PMC10320406; doi:10.1016/j.ibneur.2023.06.005)
Supplement: Supplementary file 1 — Supplementary material [file mmc1.docx]

## 6. Supplementary data

| **Supplementary table 1 \|** Formulas for the calculated parameters for the single-pool first order glucose kinetics | | |  |
| --- | --- | --- | --- |
| 1. | Concentration of D-[6,6-^2^H_2_]glucose | $\left[ {{}^{2}H}_{2}glc \right]_{t}=\left( M_{2} \right)_{t}\times\left[ glc \right]_{t}$ |  |
| 2a | Concentration curve | $\left[ {{}^{2}H}_{2}glc \right]_{t}=\left[ {{}^{2}H}_{2}glc \right]_{0}\cdot e^{-kt}$ | |
| 2b. |  | $ln\left( \left[ {{}^{2}H}_{2}glc \right]_{t} \right)=ln\left( \left[ {{}^{2}H}_{2}glc \right]_{0} \right) \kappa-\cdot t$ |  |
| 3. | Area under the curve | $AUC=\frac{\left[ {{}^{2}H}_{2}glc \right]_{0}}{k}$ |  |
| 4. | Glucose clearance rate | $GCR=\frac{{{{}^{2}H}_{2}glc}_{bolus}}{AUC}$ |  |
| 6. | Apparent volume of distribution | $V=\frac{{{{}^{2}H}_{2}glc}_{bolus}}{\left[ {{}^{2}H}_{2}glc \right]_{0}}$ |  |
| 7. | Pool size | $A=V\cdot\left[ glc \right]$ |  |
| 8. | Turnover rate = Rate of appearance | $Ra=A\cdot k$ |  |
| Relevant equations in blood glucose kinetics adapted from pharmacokinetic algorithms (van Dijk et al., 2013). [Glc] represents the blood glucose concentrations and ${(M}_{2})$ fractional contribution of the tracer. From equation 2 the fractional elimination rate (k) as well as the estimated tracer concentration at experimental time point zero ([^2^H_2_glc]_0_ was estimated in the model. All calculations were calculated using SAAM II software (Version 1.2.1 Compartmental, SAAM Institute, University of Washington). | | |  |
